# Acute myocardial infarction caused by coronary tumor embolism from uterine leiomyosarcoma: a case report

**DOI:** 10.1016/j.gore.2026.102047

**Published:** 2026-02-16

**Authors:** Yumi Shirasu, Kohei Tamura, Miki Shinohara, Yoshifumi Takahashi, Takahiro Koyanagi, Akiyo Taneichi, Yuji Takei, Hiroyuki Fujiwara

**Affiliations:** Department of Obstetrics and Gynecology, School of Medicine, Jichi Medical University, 3311-1, Yakushiji, Shimotsuke City, Tochigi 329-0498, Japan

**Keywords:** Acute myocardial infarction, Tumor embolism, Uterine leiomyosarcoma, Aspiration thrombectomy

## Abstract

**Background:**

Tumor embolism to the coronary arteries is an extremely rare cause of acute myocardial infarction (AMI). It has been reported in lung and breast cancers but not in uterine leiomyosarcoma. Herein, we present a case of AMI caused by a coronary tumor embolism from uterine leiomyosarcoma, which was successfully treated with aspiration thrombectomy.

**Case presentation:**

A 51-year-old woman with metastatic uterine leiomyosarcoma underwent surgical resection followed by adjuvant chemotherapy. During her second trabectedin cycle, she developed acute chest pain and dyspnea. Electrocardiography revealed anterior AMI. Emergency coronary angiography demonstrated complete occlusion of the left anterior descending and circumflex arteries. Thrombus aspiration completely restored coronary flow. Histopathological examination of the aspirated material confirmed leiomyosarcoma. Following aspiration, her symptoms subsided and she was discharged and prescribed cardioprotective medications. No recurrent cardiac events occurred.

**Conclusion:**

While most AMI events result from thrombotic occlusion and respond to anticoagulation, tumor emboli require mechanical intervention. Aspiration thrombectomy proved effective in this case. Clinicians should recognize that tumor embolism can induce AMI even in previously unreported cancer types, as pulmonary metastases may invade the pulmonary veins enabling tumor fragments to enter systemic circulation. Early recognition and intervention are critical for survival in these patients. Tumor embolism should be considered in the differential diagnosis of patients with cancer presenting with unexplained chest pain or dyspnea.

## Highlights

• This is the first case of acute myocardial infarction caused by coronary tumor embolism from uterine leiomyosarcoma.
• Pulmonary metastases may enable tumor fragments to enter pulmonary veins and occlude coronaries.
• Tumor embolism should be considered in patients with cancer and MI lacking typical atherosclerotic risk factors.

## Graphical abstract

**Figure:**
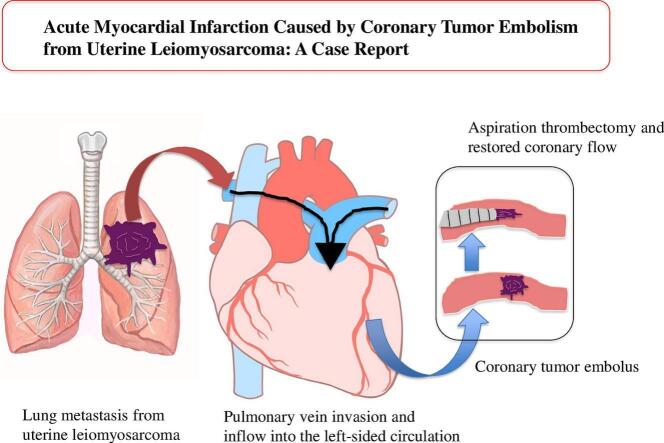

## 1 Introduction

Atherosclerotic plaque rupture is the predominant cause of acute myocardial infarction (AMI); however, coronary artery embolism has emerged as a significant but underrecognized nonatherosclerotic cause. Embolic myocardial infarction (MI) accounts for approximately 3% of acute coronary syndromes. Coronary artery embolism is most often associated with intracardiac thrombi or endovascular debris in patients with atrial fibrillation or infective endocarditis (Shibata et al. 2015). Coronary tumor embolism causing AMI is even more uncommon, and it has been sporadically reported in urothelial carcinoma (Yasui et al. 2020), lung cancer (Liu et al. 2020), and colorectal cancer (Steiner and Vojáček 2014). This rare phenomenon is of growing relevance in cardio-oncology, as coronary tumor emboli pose unique diagnostic and therapeutic challenges and emphasize the recognition of non-atherosclerotic causes of AMI in patients with cancer.

Uterine leiomyosarcoma occurs in approximately 1%–2% of uterine malignancies. It is an aggressive smooth muscle malignancy that frequently metastasizes hematogenously to distant organs, mostly the lungs (74% of cases), peritoneum (41%), liver (27%), and bone (33%) (Ahuja et al., 2017, D’Angelo and Prat, 2010, Tirumani et al., 2014). Tumor embolism associated with uterine leiomyosarcoma has been described in a few contexts, including cases with extension from the ovarian vein to the renal vein, inferior vena cava, and right atrium (Lim et al. 2015), or involving the right iliac vein and inferior vena cava (McDonald et al. 2007).

An AMI due to coronary artery occlusion from a uterine leiomyosarcoma–derived tumor embolus has not yet been reported. Herein, we describe a case in which aspiration thrombectomy for acute coronary occlusion enabled both the diagnosis and treatment of a coronary tumor embolism originating from a uterine leiomyosarcoma.

## 2 Case presentation

**Fig. 1:**
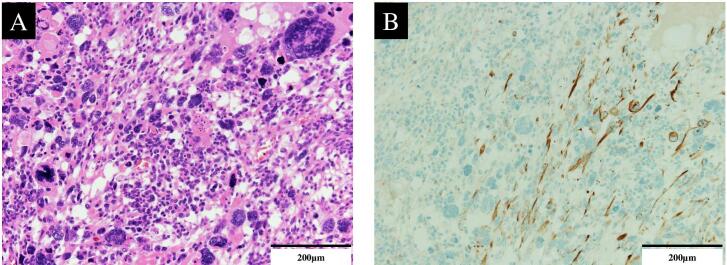
Histology of the serial sections of the uterine tumor.

A 51-year-old woman with a history of hypertension presented at our hospital with persistent fever and abdominal pain. We suspected uterine leiomyosarcoma on preoperative examination and performed emergency surgery with total abdominal hysterectomy and bilateral salpingo-oophorectomy because of an antimicrobial-resistant infection of the tumor. Pathological examination revealed a uterine leiomyosarcoma composed of cells demonstrating a spindle shape and heterogeneous nuclear atypia with positive desmin and alpha-smooth muscle actin (α-SMA) (Fig. 1A, B).

Histopathological analysis with hematoxylin and eosin staining (A) and immunohistochemistry (B) reveals spindle-shaped cells with heterogeneous nuclear atypia and positive desmin staining, consistent with smooth muscle origin (200×).

**Fig. 2:**
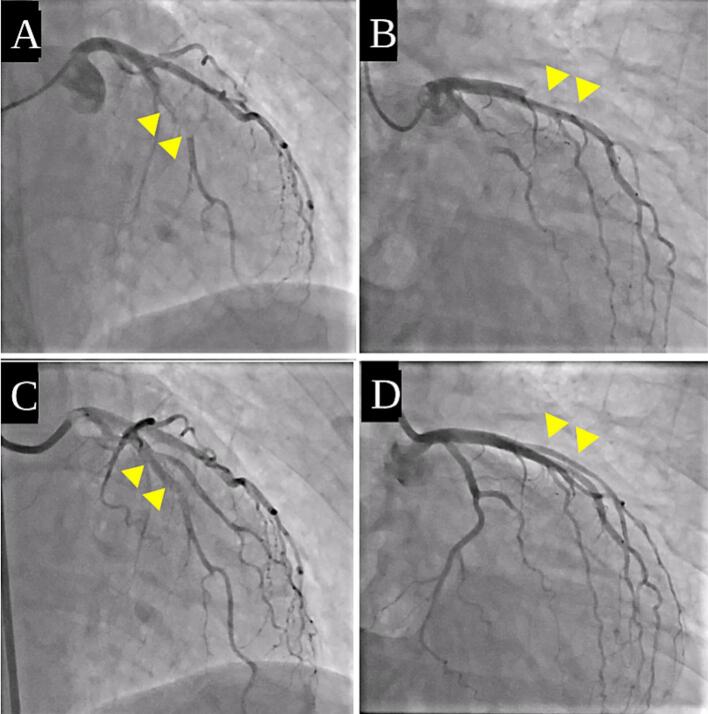
A representative coronary angiography image of the LAD at the onset of MI and after embolus retrieval.

**Fig. A.1:**
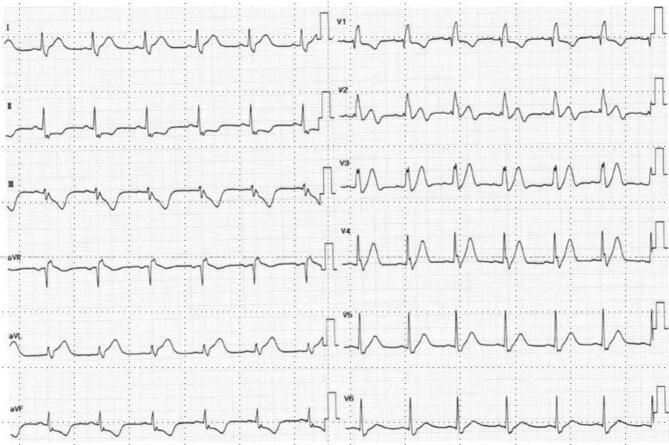
Electrocardiogram at the onset of myocardial infarction.

Lung and subcutaneous metastases of the right buttock were identified on computed tomography images, and the patient was diagnosed with stage IV leiomyosarcoma. She initially received four cycles of doxorubicin, followed by four cycles of gemcitabine plus docetaxel. Subsequently, three cycles of eribulin were administered, and oral pazopanib was continued for eight months. Despite these sequential lines of systemic therapy, the disease progressed throughout the treatment course. Finally, the patient received trabectedin chemotherapy. Two days after completing the second cycle of trabectedin, the patient developed sudden substernal chest pain and dyspnea, and she visited our emergency department. Her vital signs were: blood pressure, 94/70 mmHg; pulse rate, 82 beats/min; and peripheral oxygen saturation, 99% on room air. A 12-lead electrocardiogram showed marked ST segment elevation in leads I and aVL, with ST depression in leads II, III, and aVF (Appendix, Fig. A1). Echocardiography revealed hypokinesis of the anterior and lateral walls of the left ventricle, with a mildly reduced left ventricular ejection fraction of 50%. She underwent regular transthoracic echocardiography before each change in chemotherapy. Six months prior to the onset of MI, echocardiography demonstrated a left ventricular ejection fraction of 57% (Simpson’s method), which was a low normal, along with mild to moderate tricuspid regurgitation. Simultaneously, she reported no symptoms suggestive of heart failure, such as fatigue or chest pain. Serial electrocardiograms obtained during follow-up showed no overt abnormalities. Blood tests showed a slightly elevated troponin T level (0.018 ng/mL) and creatine phosphokinase level (336 U/L); however, creatine kinase-MB levels were within the normal range. Chest radiography revealed slight cardiomegaly, with a cardiothoracic ratio of 51% and pulmonary congestion. These findings indicated anterior AMI with heart failure, and the patient was immediately transferred to a cardiac catheterization laboratory. Coronary angiography revealed total occlusion of the left anterior descending (LAD) artery in segment #7 (Fig. 2A) and of the left circumflex (LCX) artery in segment #11 (Fig. 2B). The occlusion site had a radiolucent appearance, indicating embolic obstruction.

Aspiration thrombectomy using a catheter was performed to remove the embolic material. Specifically, a Mach 1 Coronary Guide Catheter was used as the guiding catheter, ASAHI SION blue as the guidewire, and Rebirth Pro 2 as the aspiration catheter. After aspiration, Thrombolysis in Myocardial Infarction (TIMI) grade 3 flow was restored in the LAD and LCX arteries without the need for balloon angioplasty or stenting (Fig. 2C, D).

The #7 segment (arrowhead) of the LAD (A) and #11 segment (arrowhead) of the LCX (B) are completely occluded. Flow of the #7 (C) and #11 (D) segment is fully restored.

**Fig. 3:**
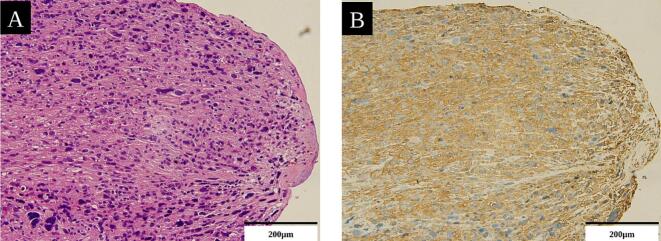
Histology of retrieved embolus with serial sections.

Gross examination of the extracted material revealed a grayish-white mass. Histopathological examination using hematoxylin and eosin staining and immunohistochemistry showed tissues closely resembling those of the primary tumor, which confirmed the diagnosis of a leiomyosarcoma embolus (Fig. 3A, B).

Hematoxylin and eosin staining (A) and immunohistochemistry (B) revealed that the tumor embolus was derived from a uterine leiomyosarcoma, with identical characteristics to the primary tumor (200×).

These findings establish that the coronary occlusion is caused by a tumor embolism from the uterine leiomyosarcoma.

After reperfusion, her symptoms rapidly improved, and no recurrent infarction was observed. Until the pathological results revealed that the embolus was tumor-related, thrombolytic therapy was performed using heparin alone. In addition, intravenous furosemide was administered for the management of heart failure, and after improvement, the treatment was transitioned to oral furosemide and spironolactone. Consequently, the patient was discharged. However, her malignancy continued to progress. No further effective treatment options were available, and she was ultimately transitioned to palliative care.

## 3 Discussion

This report demonstrated the case of an AMI caused by a tumor embolism from a uterine leiomyosarcoma, which was effectively treated with aspiration thrombectomy. To the best of our knowledge, this is the first report of an AMI caused by coronary artery occlusion because of a tumor embolus derived from a uterine leiomyosarcoma.

MI caused by coronary tumor embolism is rarely encountered in clinical practice; its exact incidence has not been reported, and it has only been documented in isolated case reports (Liu et al., 2020, Saleh et al., 2024, Steiner and Vojáček, 2014, Yasui et al., 2020). Lung carcinoma is the most common cause of malignant coronary embolism, likely owing to its propensity to invade the pulmonary veins and seed tumor fragments into the left heart circulation. It causes AMI because of the direct tumor extension into the left atrium, with subsequent embolization to the coronary arteries in some cases (Liu et al. 2020). Gastric adenocarcinoma and other gastrointestinal tumors have also been associated with tumor thromboembolism, particularly in the pulmonary vasculature; however, systemic arterial embolization from gastric cancer is exceedingly rare (Roberts et al. 2003). Urothelial carcinoma offers another mechanism; a recent cardio-oncology case report described an AMI from a tumor embolus that crossed a patent foramen ovale into the coronary arteries (Yasui et al. 2020). Therefore, while, theoretically, any malignancy with intravascular access can embolize to the coronary arteries, such events remain exceptionally uncommon and are typically observed in singular case reports.

More broadly, coronary tumor embolism can be viewed as one manifestation of arterial tumor embolization, a rare but clinically important cause of acute arterial occlusion. A recent systematic review of published case reports found that lung cancer and atrial myxoma were among the most frequent sources, and that clinical presentations most commonly included ischemic stroke, followed by MI and acute limb ischemia. Reported predisposing factors include advanced stage disease, a friable or irregular tumor surface, and an anatomic substrate that allows direct access to the systemic arterial circulation—particularly thoracic tumors with invasion of the pulmonary veins and/or the left atrium—as well as tumor fragmentation triggered by surgical manipulation (Hussain 2022). In addition, a large pathologic series with a comprehensive literature review emphasized that, despite the rarity of arterial neoplastic emboli (<1% of thromboemboli), embolic events are the sentinel manifestation of an underlying malignancy; therefore, they support routine histopathologic evaluation of retrieved embolic material when the clinical context is suggestive (Bois et al. 2018).

The mechanism by which uterine leiomyosarcoma led to coronary embolism in our patient could be attributed to known patterns of metastatic spread. Given her history of pulmonary metastases, one plausible pathway is that a tumor fragment from a lung metastatic lesion invaded the pulmonary vein and entered the systemic circulation, similar to the mechanism described in primary lung cancers that shed emboli into the left heart (Liu et al. 2020). The event occurred after chemotherapy; therefore, treatment-induced tumor necrosis or enhanced vascular invasion may have also contributed to embolus formation. Alternatively, microscopic tumor emboli may have traversed a transient right-to-left shunt; however, no such defects were reported in this patient. This report underscores the need to consider tumor embolism as a cause of AMI in patients with cancer who present with sudden chest pain or dyspnea. In our case, clinical suspicion of tumor embolism was heightened because the patient had an active malignancy and lacked conventional atherosclerotic risk factors, such as atrial fibrillation and dyslipidemia, making MI caused by conventional atherosclerotic plaque less likely. The clinical context of our patient—progressive metastatic disease with pulmonary involvement and recent systemic chemotherapy—overlaps with these proposed risk factors for arterial tumor embolization (Hussain 2022).

There are no standardized guidelines for the management of coronary tumor embolisms. Zhao et al. suggested that aspiration thrombectomy is superior to stenting for obtaining favorable reflow with tumor emboli (Zhao et al. 2023). Stent implantation for tumor emboli may be suboptimal because tumor tissue can proliferate and cause re-occlusion or be displaced distally, exacerbating downstream obstruction. Moreover, stenting prevents retrieval of embolic material and histopathological diagnosis. Therefore, aspiration thrombectomy is preferred when tumor embolism is suspected (Raphael et al. 2018). Our report highlights the critical role of aspiration thrombectomy in the management and diagnosis of tumor embolism. In contrast to standard thrombotic occlusions, obstructing tumor plugs do not respond to fibrinolysis, anticoagulation, or antiplatelet therapies. Prompt mechanical removal is often the only method for restoring perfusion. In our patient, aspiration of the occlusive mass immediately re-established coronary flow and enabled histopathological confirmation of leiomyosarcoma, which could not be achieved by angiography alone. The management of MI caused by tumor embolism requires multidisciplinary collaboration. When patients with cancer without typical atherosclerotic risk factors present with chest pain or dyspnea, clinicians should maintain awareness of the potential for tumor embolism.

This case had some limitations. As a single case report, we could not establish definitive causation or generalizability. The proposed mechanism, which involved pulmonary vein invasion, remains speculative without autopsy confirmation. Additionally, long-term follow-up data are lacking owing to the patient’s progressive malignancy and transition to palliative care.

## 4 Conclusion

Aspiration thrombectomy is an effective therapeutic approach for treating tumor emboli. Even in malignancies with no prior reports of MI, such as uterine leiomyosarcoma, tumor embolism-related MI can occur. Clinicians should recognize that tumor emboli may reach the coronary arteries via the pulmonary veins and cause MI in patients with lung metastases. Early recognition and mechanical intervention are critical for patient survival.

## 5 Consent

Written informed consent was obtained from the patient for the publication of this case report and its accompanying images. A copy of the written consent is available for review by the Editor-in-Chief upon request.

## CRediT authorship contribution statement

**Yumi Shirasu:** Writing – original draft, Investigation. **Kohei Tamura:** Writing – original draft, Investigation, Conceptualization. **Miki Shinohara:** Writing – review & editing. **Yoshifumi Takahashi:** Writing – review & editing. **Takahiro Koyanagi:** Writing – review & editing. **Akiyo Taneichi:** Writing – review & editing. **Yuji Takei:** Writing – review & editing. **Hiroyuki Fujiwara:** Supervision.

## Declaration of competing interests

The authors declare that they have no known competing financial interests or personal relationships that could appear to have influenced the work reported in this paper.

## Appendix

ST segment elevation is observed in leads I and aVL, with ST segment depression in leads II, III, and aVF.
